# Significance of RNA N6-methyladenosine regulators in the diagnosis and subtype classification of coronary heart disease using the Gene Expression Omnibus database

**DOI:** 10.3389/fcvm.2023.1185873

**Published:** 2023-10-19

**Authors:** Yu Jiang, Yaqiang Pan, Tao Long, Junqing Qi, Jianchao Liu, Mengya Zhang

**Affiliations:** ^1^ Department of Cardiovascular Surgery, Yan'an Hospital affiliated to Kunming Medical University, Yunnan, China; ^2^ Department of Cardiothoracic Surgery, Affiliated People's Hospital of Jiangsu University, Zhenjiang, China; ^3^ Department of Cardiology, the Affiliated Suzhou Hospital of Nanjing Medical University, Suzhou Municipal Hospital, Gusu School of Nanjing Medical University, Suzhou, China

**Keywords:** coronary heart disease, m6A RNA methylation regulator, consensus clustering, Gene Expression Omnibus database, risk prediction model, immune cell infiltration

## Abstract

**Background:**

Many investigations have revealed that alterations in m6A modification levels may be linked to coronary heart disease (CHD). However, the specific link between m6A alteration and CHD warrants further investigation.

**Methods:**

Gene expression profiles from the Gene Expression Omnibus (GEO) databases. We began by constructing a Random Forest model followed by a Nomogram model, both aimed at enhancing our predictive capabilities on specific m6A markers. We then shifted our focus to identify distinct molecular subtypes based on the key m6A regulators and to discern differentially expressed genes between the unique m6A clusters. Following this molecular exploration, we embarked on an in-depth analysis of the biological characteristics associated with each m6A cluster, revealing profound differences between them. Finally, we delved into the identification and correlation analysis of immune cell infiltration across these clusters, emphasizing the potential interplay between m6A modification and the immune system.

**Results:**

In this research, 37 important m6Aregulators were identified by comparing non-CHD and CHD patients from the GSE20680, GSE20681, and GSE71226 datasets. To predict the risk of CHD, seven candidate m6A regulators (CBLL1, HNRNPC, YTHDC2, YTHDF1, YTHDF2, YTHDF3, ZC3H13) were screened using the logistic regression model. Based on the seven possible m6A regulators, a nomogram model was constructed. An examination of decision curves revealed that CHD patients could benefit from the nomogram model. On the basis of the selected relevant m6A regulators, patients with CHD were separated into two m6A clusters (cluster1 and cluster2) using the consensus clustering approach. The Single Sample Gene Set Enrichment Analysis (ssGSEA) and CIBERSORT methods were used to estimate the immunological characteristics of two separate m6A Gene Clusters; the results indicated a close association between seven candidate genes and immune cell composition. The drug sensitivity of seven candidate regulators was predicted, and these seven regulators appeared in numerous diseases as pharmacological targets while displaying strong drug sensitivity.

**Conclusion:**

m6A regulators play crucial roles in the development of CHD. Our research of m6A clusters may facilitate the development of novel molecular therapies and inform future immunotherapeutic methods for CHD.

## Introduction

Coronary heart disease (CHD) is a leading cause of death and disability worldwide, despite significant progress in the areas of detection and treatment (1). It is imperative and essential to develop more effective diagnostic and therapeutic procedures, which would be helped by a deeper understanding of the underlying mechanisms (2). RNA methylation, particularly N6-methyladenosine (m6A), is the most widespread epigenetic modification of RNA nucleotides. Current epigenetics research has yielded numerous noteworthy advances. In eukaryotic species, m6A accounts for over 97.4% of internal RNA modification and is extremely conservative.

Adenosine methyltransferases (the writers), m6A-binding proteins (the readers), and m6A demethylating enzymes (the erasers) are the three main components of m6A, which was first discovered in the 1970s (3, 4). Given the vital role m6A plays in many fundamental biological processes, it has become a hot topic recently. m6A plays a role in a wide range of diseases, including cancer, type 2 diabetes mellitus, obesity, and more through its regulation of mRNA stability and homeostasis. Dysregulation of m6A at the phenotypic level has been linked to carcinogenesis (5). Obesity and other metabolic disorders are linked to FTO protein, which is essential for the regulation of genome-wide m6A modification in mRNA (6). Although m6A regulators have been implicated in CHD, their precise roles remain unknown.

We analyzed the roles of m6A regulators in the identification and categorization of CHD subtypes using the GSE20680, GSE20681, and GSE71226 datasets. Seven regulators of the m6A gene were selected to assess CHD incidence. On their basis, a risk model and nomogram were developed, from which CHD patients might derive significant clinical benefit. Moreover, two different m6A clusters were identified. The relationship between the two clusters and the immune cell infiltration and possible medication selection was strong. In conclusion, a deeper knowledge of m6A processes will enhance the development of novel molecular therapies and guide future immunotherapy tactics for CHD.

## Materials and methods

### Date acquisition

The analyzed data were downloaded from the Gene Expression Omnibus (GEO) database (https://www.ncbi.nlm.nih.gov/geo/) (7, 8). The GSE71226 dataset included 3 patients with CHD and 3 without; the GSE20680 dataset included 143 patients with CHD and 52 without; and the GSE20681 dataset included 99 patients with CHD and 99 without. The data have been gathered and analyzed. Then, using a difference analysis between CHD and non-CHD patients, we were able to identify a total of 37 m6A regulators. These comprised twenty-three distinct regulators: eleven writers (WTAP, VIRMA, METTL3, METTL14, ZC3H13, RBM15B, CBLL1, RBM15, METTL16, ZCCHC4, and PCIF1), two erasers (FTO and ALKBH5), and ten readers (HNRNPA2B1, HNRNPC, RBMX, IGF2BP1, IGF2BP2, IGF2BP3, YTHDF1, YTHDF2, YTHDF3, YTHDC1, and YTHDC2), with some regulators such as METTL16 and PCIF1 possessing dual functions.

### Construction of the nomogram model

In this study, we utilized the “glm function” of the “glmnet” package in the R statistical software to develop a univariate and multivariate logistic regression-based training model for predicting the occurrence of CHD. The threshold for significant genes in univariate logistic regression was set at *P*-value <0.5. We then included the candidate modulators in the risk prediction model, and constructed a prediction formula based on normalized gene expression values weighted by coefficients. A nomogram was developed using the candidate regulators to predict the prevalence rates of CHD. The calibration curve measured the congruence between predictions and reality. A decision curve analysis (DCA) was conducted to evaluate the clinical value of the model's predictions for individual patients.

### Identification of molecular subtypes based on the significant m6A regulators

Consensus clustering is used to validate the clustering rationale based on resampling, as well as to determine the number of clusters. We identified distinct m6A clusters according to the crucial m6A regulators using the “ConsensusClusterPlus” package in R (9).

### Identification of differentially expressed genes between distinct m6A clusters

We used the “limma” package to identify differentially expressed genes (DEGs) between m6A clusters (10). The absolute value of log2FC needed to be greater than 1.5, and the *P*-value needed to be less than 0.05 for the screening criterion to be satisfied. Both a heat map and a volcano plot were produced.

### Analysis of biological characteristics between distinct m6A clusters

Gene ontology (GO) includes biological processes (BP), molecular functions (MF), and cellular components (CC), and is commonly used in enrichment analysis to interpret gene lists (11). The Kyoto Encyclopedia of Genes and Genomes (KEGG) is a comprehensive pathway-oriented knowledge base, containing 15 related databases covering 3,982 organisms (12). We performed GO and KEGG analysis using the “clusterProfiler” package (13) in R software to better understand the potential mechanism of the DEGs involved in CHD. We set an FDR < 0.05 as the screening criterion. To determine which biological processes varied significantly between the clusters, we conducted a Gene Set Enrichment Analysis (GSEA). GSEA is often used to evaluate the statistically significant distinction between two biological states (14). To conduct GSEA, we used the gene sets c2.cp.kegg.v7.4.symbols.gmt and c5.go.v7.2.symbols.gmt from the Molecular Signature Database (MSigDB). *P*-values less than 0.05 were considered statistically significant.

### Construction of protein-protein interaction networks (PPI)

We used the Search Tool for the Retrieval of Interacting Genes (STRING) database (15), a resource for known and predicted PPI, to construct a PPI network for DEGs. In our study, DEGs with a total score greater than 400 were selected to construct PPI networks, which were visualized using Cytoscape (v3.7.2) (16). Molecular Complex Detection (MCODE) (17) detects densely connected regions in large PPI networks that may represent molecular complexes, the sub-networks with highest score were excavated as critical sub-networks. ClueGO (18) was used to annotate the sub-networks of genes.

### Identification and correlation analysis of immune cell infiltration between distinct m6A clusters

We used a variant of GSEA called single-sample GSEA (ssGSEA) to quantify the abundance of each of 28 different types of immune cells in CHD tissue samples (19). CIBERSORT is an algorithm developed for microarray data that leverages a reference matrix generated from purified cell populations. We utilized the CIBERSORT algorithm in R software to estimate the abundance of 22 immune cell types. Boxplots were used to illustrate the immune cell composition across different m6A clusters. The Wilcoxon test was applied to compare the proportions of immune cells, and results with a *P*–value <0.05 were considered significant.

### Drug sensitivity analysis and disease enrichment analysis

To explore potential therapeutic strategies, the sensitivity of candidate m6A regulators to drugs was predicted. The drug-gene interaction database (DGIdb) was used to identify drugs that target the identified m6A regulators. Additionally, disease enrichment analysis of the candidate m6A regulators was performed using the DisGeNET database to understand the potential implications of these regulators in other diseases.

In conclusion, the comprehensive analysis of m6A regulators in our study provides insights into the molecular mechanisms of CHD and may contribute to the development of novel therapeutic strategies. The constructed nomogram and identified molecular subtypes may serve as valuable tools for personalized treatment of CHD.

### Sample collection and QRT-PCR analysis

Our samples were obtained from Affiliated People's Hospital of Jiangsu University. These samples were collected after strict adherence to ethical guidelines and approval by the hospital ethics committee. All participants signed an informed consent form after being informed of the purpose and procedure of the study. We collected 4 normal and CHD blood samples according to a predetermined standard protocol. First, we asked participants to have their blood collected in the morning on an empty stomach. Next, we performed the blood collection under strictly sterile conditions using anticoagulation-free tubes and placed the samples in the refrigerator immediately after the blood collection.

Total RNA was extracted from 4 normal and 4 CHD blood samples using TRIzol Reagent (Life Technologies-Invitrogen, Carlsbad, CA, USA), in accordance with the manufacturer's guidelines. The concentration and purity of the RNA solution were assessed using a NanoDrop 2000FC-3100 nucleic acid protein quantifier (Thermo Fisher Scientific, Waltham, MA, USA). The RNA was then reverse-transcribed to cDNA using the SureScript-First-strand-cDNA-synthesis-kit (Genecopoeia, Guangzhou, China) before proceeding with quantitative real-time PCR (qRT-PCR). The qRT-PCR experiment consisted of 3 µl of reverse transcription product, 5 µl of 5BlazeTaq qPCR Mix (Genecopoeia, Guangzhou, China), and 1 µl each of forward and reverse primers. Following an initial denaturation at 95°C for 1 min, 40 cycles of incubation were conducted at 95°C for 20 s, 55°C for 20 s, and 72°C for 30 s. The sequence information for all primers produced by Servicebio (Wuhan, China) is provided in Table 1. The GAPDH gene was used as an internal reference, and the 2^−ΔΔCt^ method was employed to calculate the relative expression of the seven diagnostic genes. The experiment was repeated three times. Comparisons of the seven diagnostic m6A regulators between normal and CHD samples were made using paired *t*-tests and GraphPad Prism V6 (GraphPad Software, La Jolla, CA, USA). The primer sequences for 4 m6A-related genes were shown in Table 1.

**Table 1:** The primer sequences for 4 m6A-related genes and GAPDH.

Gene	Forward	Reverse
GAPDH	5′-AGCCACATCGCTCAGACAC-3′	5′-GCCCAATACGACCAAATCC-3′
YTHDF2	5′-AGCCCCACTTCCTACCAGATG-3′	5′-TGAGAACTGTTATTTCCCCATGC-3′
YTHDF3	5′-TCAGAGTAACAGCTATCCACCA-3′	5′-GGTTGTCAGATATGGCATAGGCT-3′
HNRNPC	5′-GATCTTCAGCTACATTTTCGGC-3′	5′-TGGAGCGAGGATCTGTCTTG-3′
ZC3H13	5′-TCTGATAGCACATCCCGAAGA-3′	5′-CAGCCAGTTACGGCACTGT-3′

### Statistical analysis

All statistical analyses were performed using R 4.1.1. Student's *t*-tests were used for continuous variables with a normal distribution, Wilcoxon rank-sum tests for those with a non-normal distribution, and chi-square or Fisher's Exact tests (where appropriate) for categorical variables. Pearson correlation analysis was utilized to assess the correlation between genes. *P*-values were two-tailed, with *P-*value <0.05 indicating statistical significance.

## Results

### Landscape of the m6A-related regulators in coronary heart disease

We removed batch effects of selected GEO data sets and an integrated data set containing 245 CHD and 154 non-CHD patients was acquired (Figure 2). Using the “limma” package in R, we compared the expression levels of 37 m6A regulators in CHD and non-CHD samples. Of these, 29 were deemed to be significant m6A regulators (ALKBH3, FTO, HNRNPA2B1, IGF2BP1, IGF2BP2, IGF2BP3, CPFS6, EIF3A, LRPPRC, ALKBH5, CBLL1, FMR1, HNRNPC) and were selected and displayed with a heat map and box plot. We discovered that patients with CHD had increased expression of the m6A regulators NXF1, ALKBH, HNRNPC, FMR1, HNRNPA2B1, WTAP, YTHDC1, and YTHDF3, whereas individuals without CHD had lower expression of the other important m6A regulators (Figures 3A,B). The “RCircos” software tool allowed for the visualization of chromosomal sites such as 29 m6A regulators (20).

**Figure 1:**
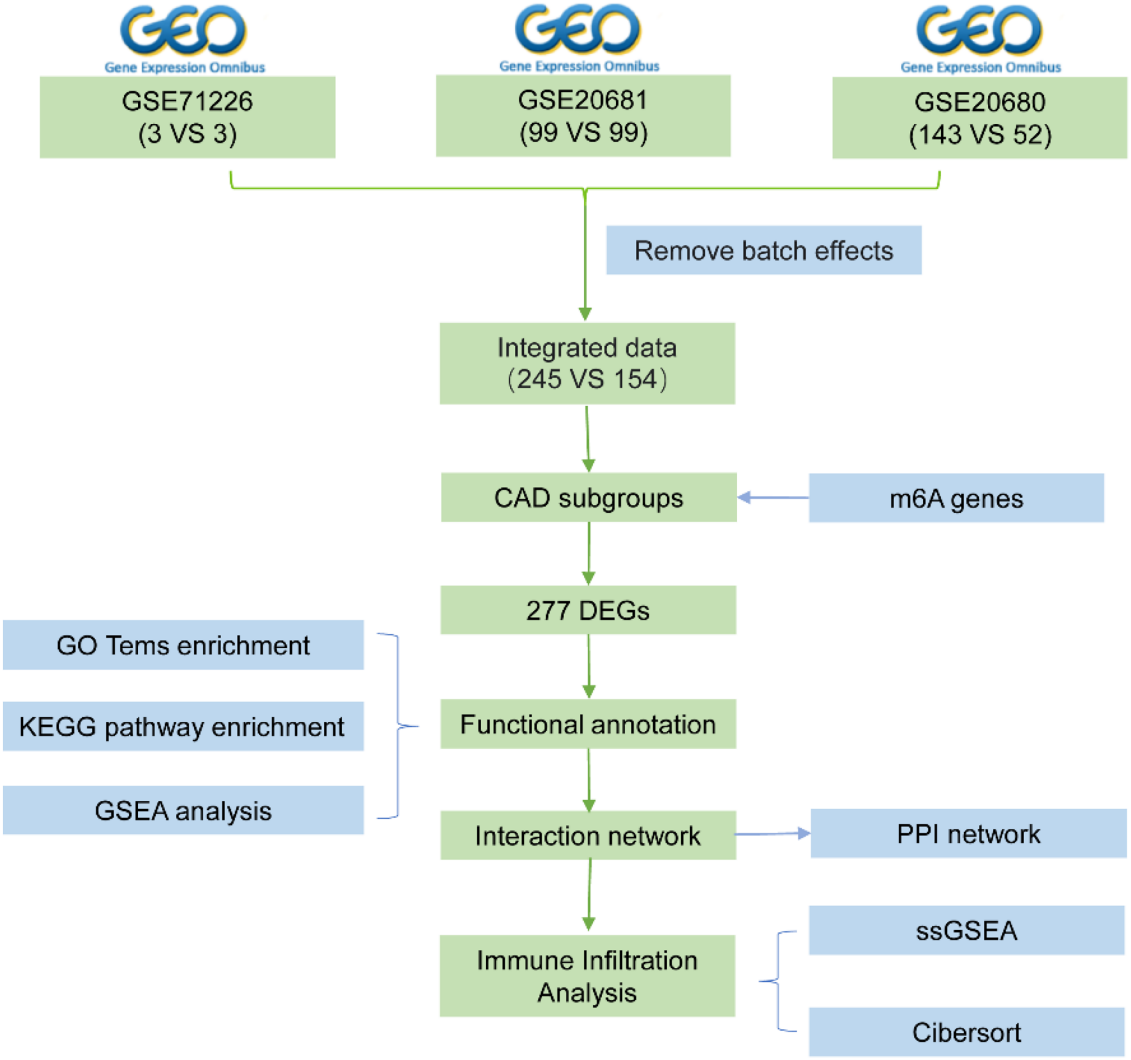
Flow chart.

**Figure 2:**
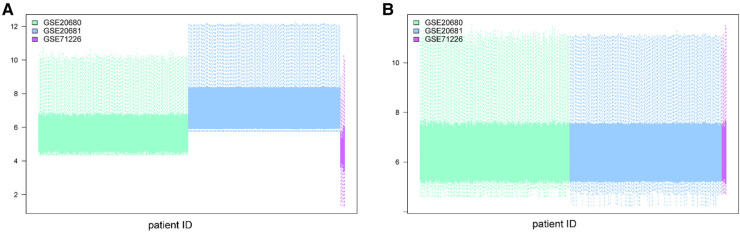
Batch effect removal using the “limma” package in R. (**A**) Datasets before batch effect removal. (**B**) Datasets after batch effect removal.

**Figure 3:**
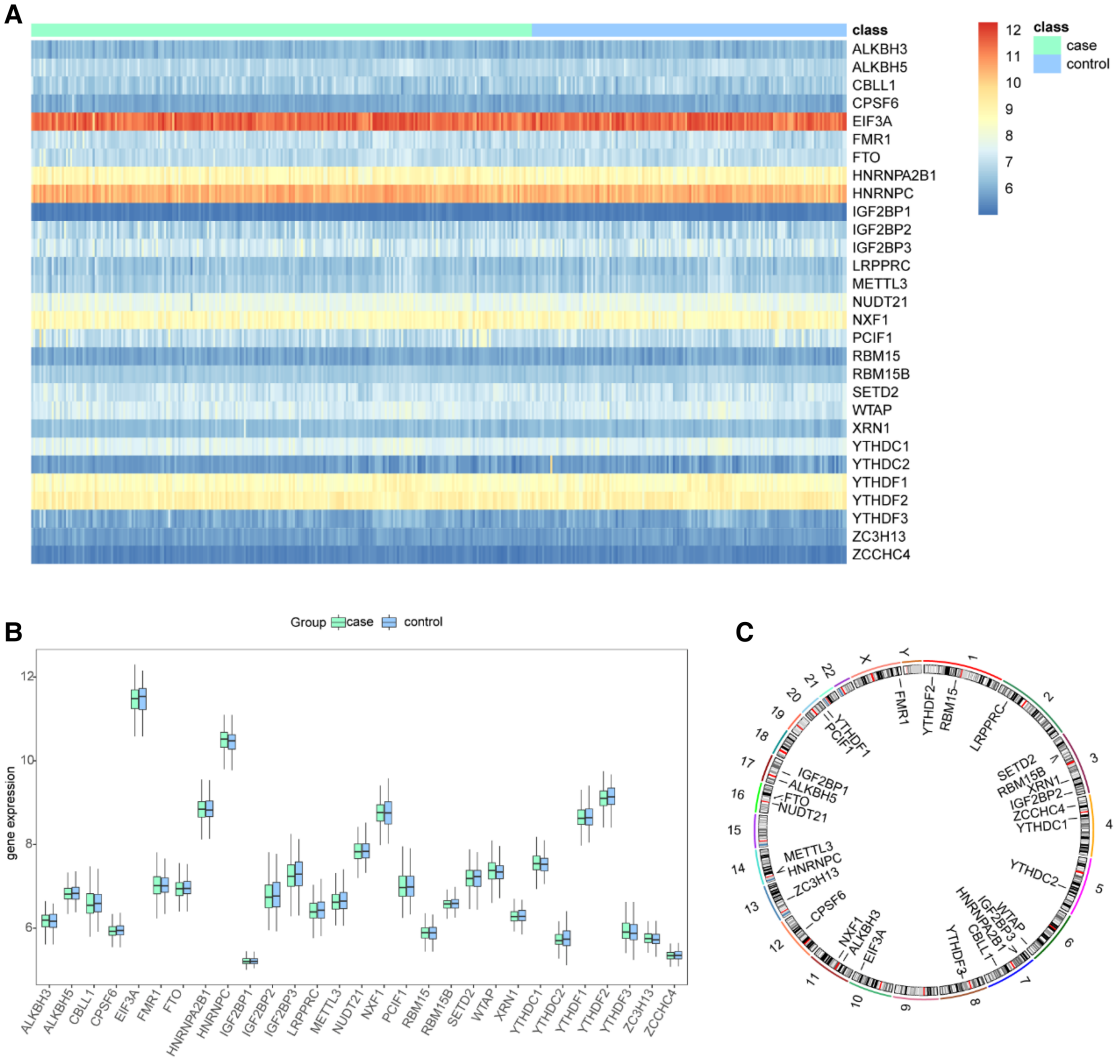
Landscape of the m6A-related regulators in CHD. (**A**) Expression heat map of the 29 m6A regulators in CHD and non-CHD patients. (**B**) Differential expression histogram of the 29 m6A regulators identified between CHD and non-CHD patients. (**C**) Chromosomal positions of the 29 m6A regulators.

### Correlation between writers and erasers in CHD

**Figure 4:**
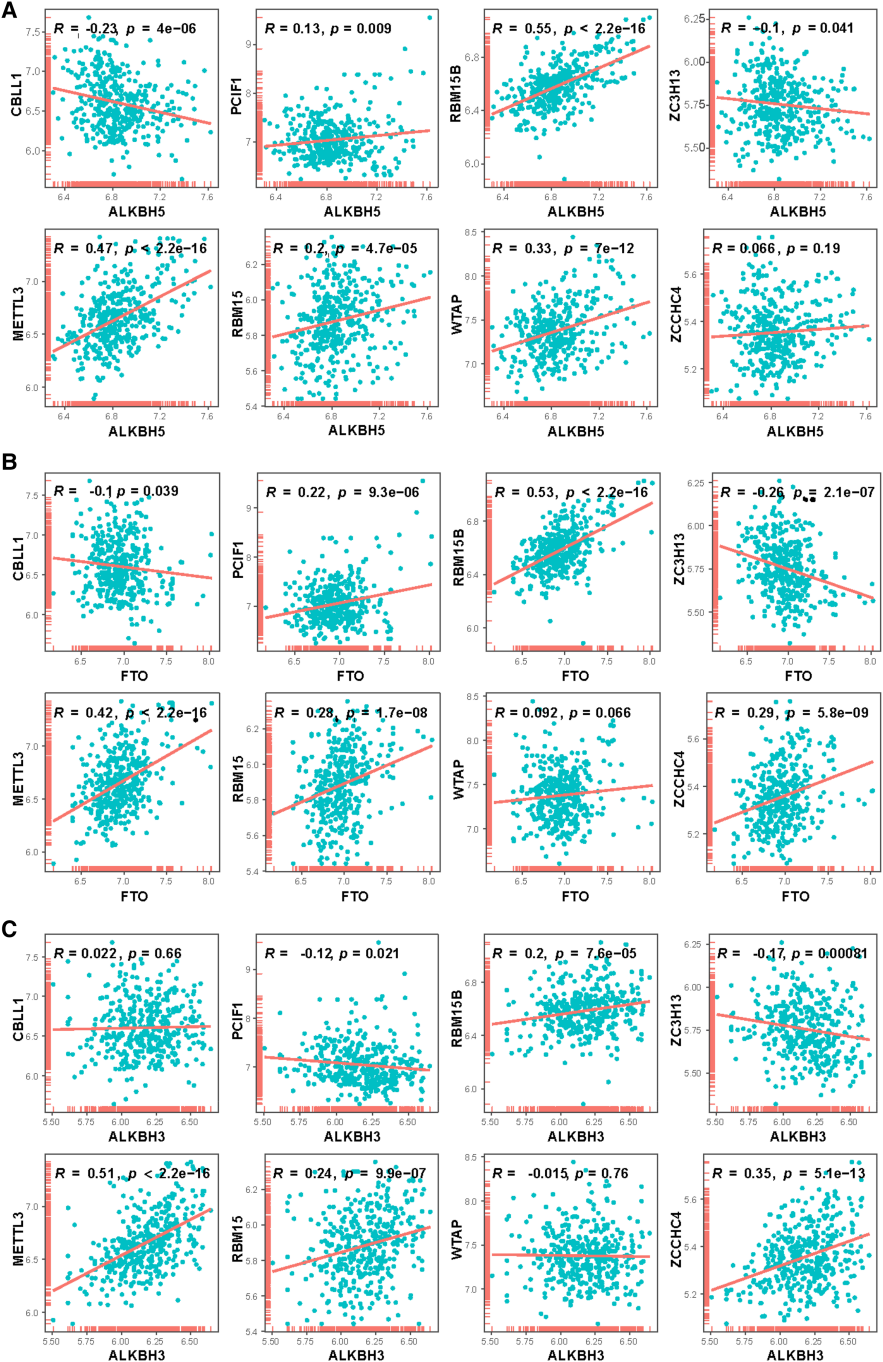
Correlation between writers and erasers in CHD patients. 8 writer genes and 3 eraser genes were analyzed. Horizontal axis represents the expressions of erasers, vertical axis represents the expressions of writers. (**A**) ALKBH5, (**B**) FTO, (**C**) ALKBH3.

Correlation analysis was performed to explore at the connection between writer and eraser genes to see if writer gene expression in CHD was linked to eraser gene expression. We found a significant positive correlation between ALKBH5 expression levels and those of RBM15B, METTL3, and WTAP in patients with CHD (Figure 4A). In patients with CHD, a significant positive correlation was observed between the expression of FTO and that of RBM15B and METTL3 (Figure 4B), and the expression of ALKBH3 also demonstrated a positive connection with METTL3 and ZCCHC4 (Figure 4C), and there were significant relationships between the ALKBH3 and RBM15B, METTL3, RBM15 and ZCCHC4. This might suggest the existence of a systemic methylation regulation pattern. It could indicate that during the development of CHD, certain important biological processes or signaling pathways might be regulated through methylation.

Furthermore, the discovery of positive correlation could potentially reveal some hidden disease biomarkers or therapeutic targets. If these positively correlated methylated genes are functionally linked, there might be one or several key genes or pathways leading these epigenetic changes, which could provide opportunities for the development of novel treatment methods.

### Construction of risk prediction models

**Figure 5:**
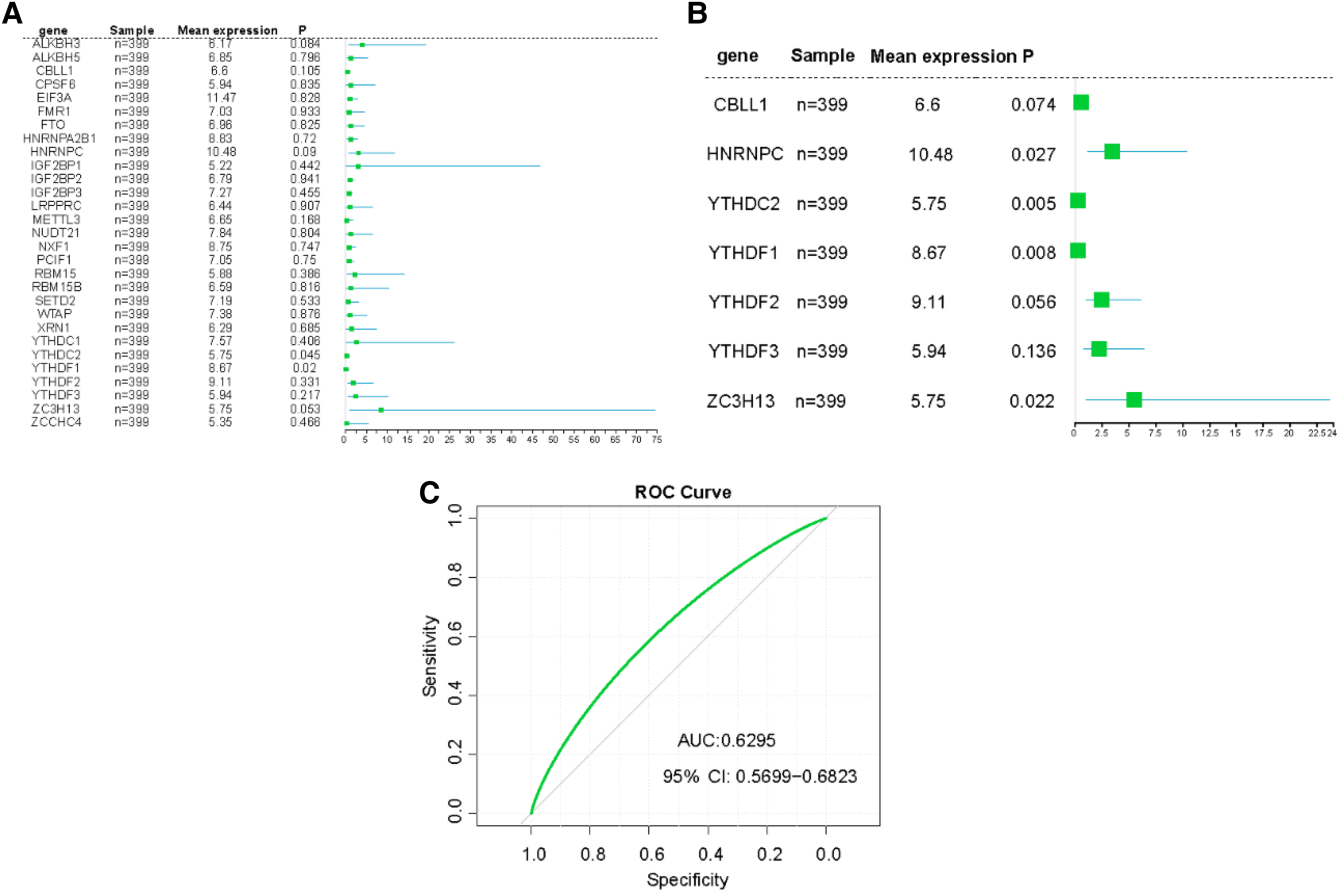
Construction of risk prediction models. (**A**) The RF model of m6A related genes in CHD patients. (**B**) The RF model of 7 m6A related genes with greater impact in CHD patients. (**C**) ROC curve the CHD predictive model.

In our detailed investigation of genes associated with CHD, we first applied univariate logistic regression to sift out the most significant genes (*P*-value <0.5). Through this procedure, we calculated predictive scores for all patients by multiplying the gene expression values by their respective coefficients (Figure 5A). To further refine our gene selection, we adopted a multivariate logistic regression by using 14 candidate genes. Recognized for its exceptional performance in handling complex datasets and feature selection, the application of multivariate logistic regression ultimately led us to single out seven genes. These genes demonstrated impressive performance in our predictive model (Figure 5B). To evaluate the predictive capability of our model, we employed a receiver operating characteristic (ROC) curve and computed the area under the curve (AUC). The high AUC value in our model clearly attests to its effectiveness in identifying CHD cases (Figure 5C). These results strongly suggest that these seven genes may play pivotal roles in the onset and progression of CHD. We hope to delve deeper into understanding the mechanisms of these genes in CHD and explore their potential as diagnostic markers or therapeutic targets.

### PPI networks of m6A regulators

**Figure 6:**
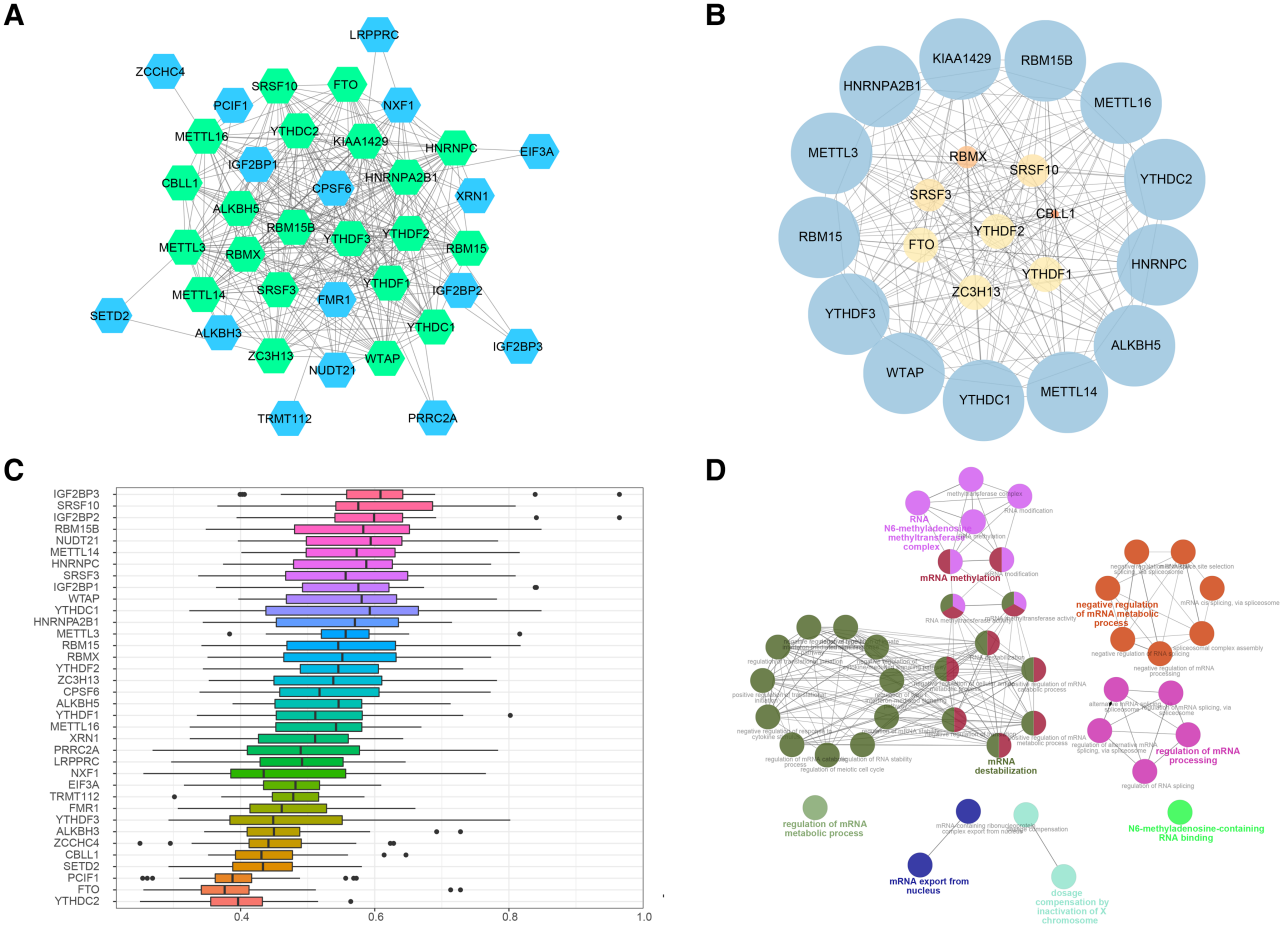
PPI networks of m6A regulators. (**A**) Protein-protein interaction networks of m6A regulators, green nodes refered to the genes included in the 37 regulators. (**B**) Sub-networks of m6A regulators, size of node indicated the MCODE score. (**C**) Functional similarity analysis of m6A regulators in PPI sub-networks. (**D**) Functional enrichment analysis of m6A regulators m6A regulators in PPI sub-networks.

To further ascertain the potential functions of the 37 m6A methylation regulators, we established PPI networks and visualized these networks using Cytoscape (Figure 6A). This resulted in the identification of 300 interactions involving 37 genes. Importantly, we discovered that HNRNPA2B1 and YTHDF3 were closely linked to 27 other m6A regulators, while HNRNPC, KIAA1429, METTL3, YTHDF1, and YTHDF2 were intimately associated with 26 m6A regulators, emphasizing their potential significance in the m6A regulation network. We further extracted sub-networks using the MCODE algorithm, which incorporated 21 genes (Figure 6B). Among these, KIAA1429, HNRNPA2B1, METTL3, RBM15, and YTHDF3 emerged as the top five scoring genes, underlining their central roles within the network. Interestingly, we observed a substantial degree of functional similarity among the 21 genes m6A regulators (Figure 6C), suggesting the existence of coordinated and interconnected regulatory mechanisms. To comprehend the functional implications of these sub-networks, we performed a ClueGO functional enrichment analysis. This analysis revealed that the genes within the sub-networks were significantly enriched in functions related to N6-methyladenosine-containing RNA binding, the RNA N6-methyladenosine methyltransferase complex, regulation of mRNA metabolic process, mRNA export from the nucleus, and dosage compensation by inactivation of the X chromosome (Figure 6D).

These findings provide invaluable insights into the complex network of interactions and regulatory roles of key m6A regulators in CHD. This sets a solid foundation for further investigations into the functional impacts of these regulators and their potential as therapeutic targets for CHD.

### Construction of the nomogram model

**Figure 7:**
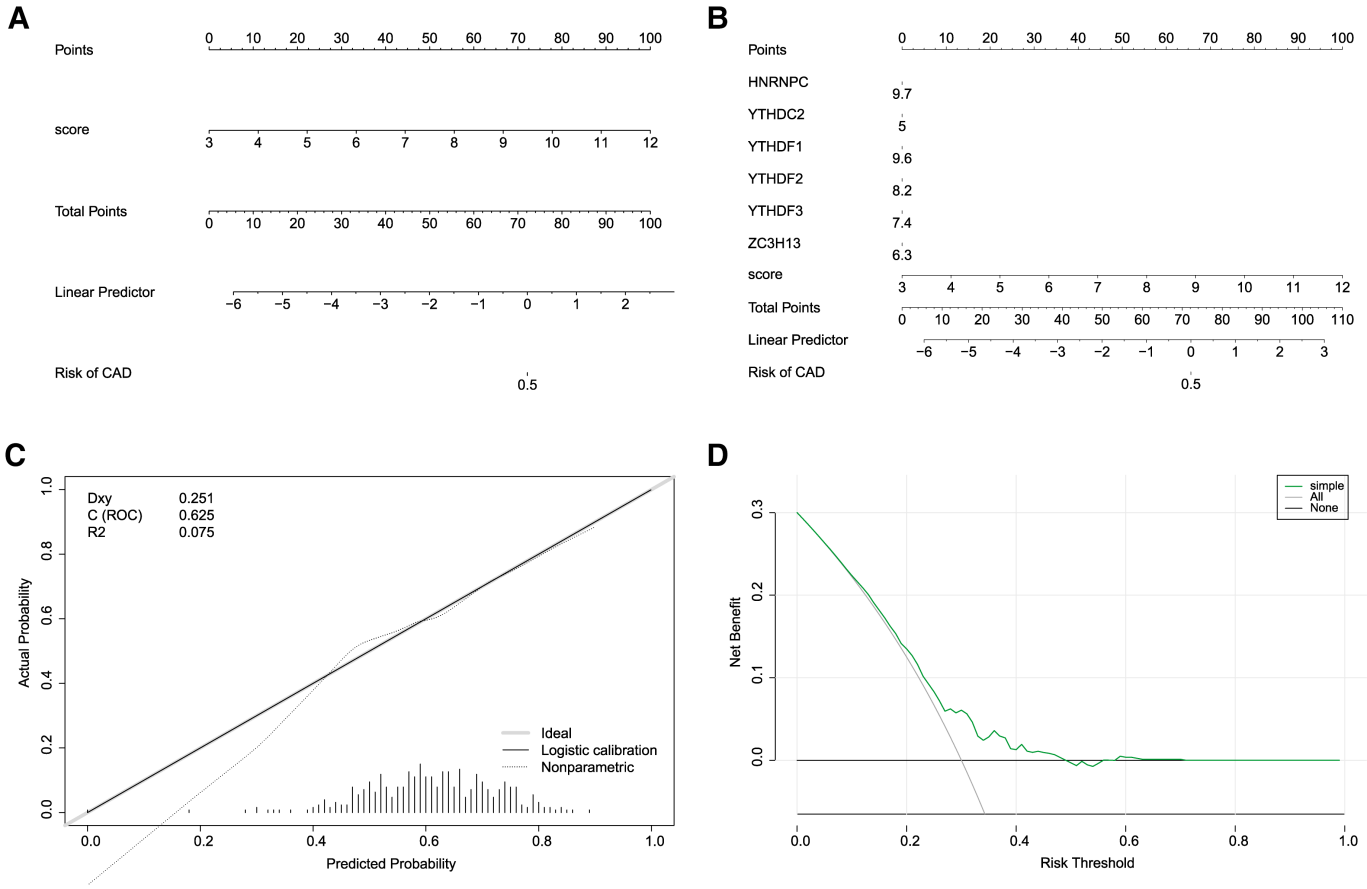
Construction of nomogram models. (**A,B**) Construction of the nomogram model based on the 7 candidate RNA N6-methyladenosine regulators. (**C**) Predictive ability of the nomogram model as revealed by the calibration curve. (**D**) Decisions based on the nomogram model may benefit CHD patients.

Building on the findings from our gene selection and predictive modeling, we took one step further to construct a nomogram model, with the goal to estimate the incidence of CHD based on the identified m6A regulators. To this end, we utilized the “rms” package in R to develop the nomogram model, incorporating the expression profiles of the five identified m6A regulators as key variables (Figures 7A,B). The validity and accuracy of our nomogram were evaluated through calibration curves. These curves showed that the predictions made by our nomogram were remarkably accurate, demonstrating a close correspondence between the predicted and actual observed incidence of CHD (Figure 7C). Moreover, we conducted a DCA to evaluate the clinical utility of our nomogram. Remarkably, the decision curve revealed that the use of our nomogram in clinical decision-making could be beneficial for CHD patients, as the green line in the DCA curve (representing the nomogram) stayed consistently above the grey and black lines (representing the treat-all-patients and treat-none scenarios, respectively) across the entire range of threshold probabilities from 0 to 1 (Figure 7D).

In conclusion, our nomogram model, underpinned by five key m6A regulators, offers a promising and effective tool for predicting the incidence of CHD. This finding has the potential to inform clinical decision-making, aiding in the early detection and intervention of CHD. However, further studies are needed to confirm these findings and explore the precise roles of these m6A regulators in the pathogenesis of CHD.

### Two distinct m6A clusters identified by significant m6A regulators

**Figure 8:**
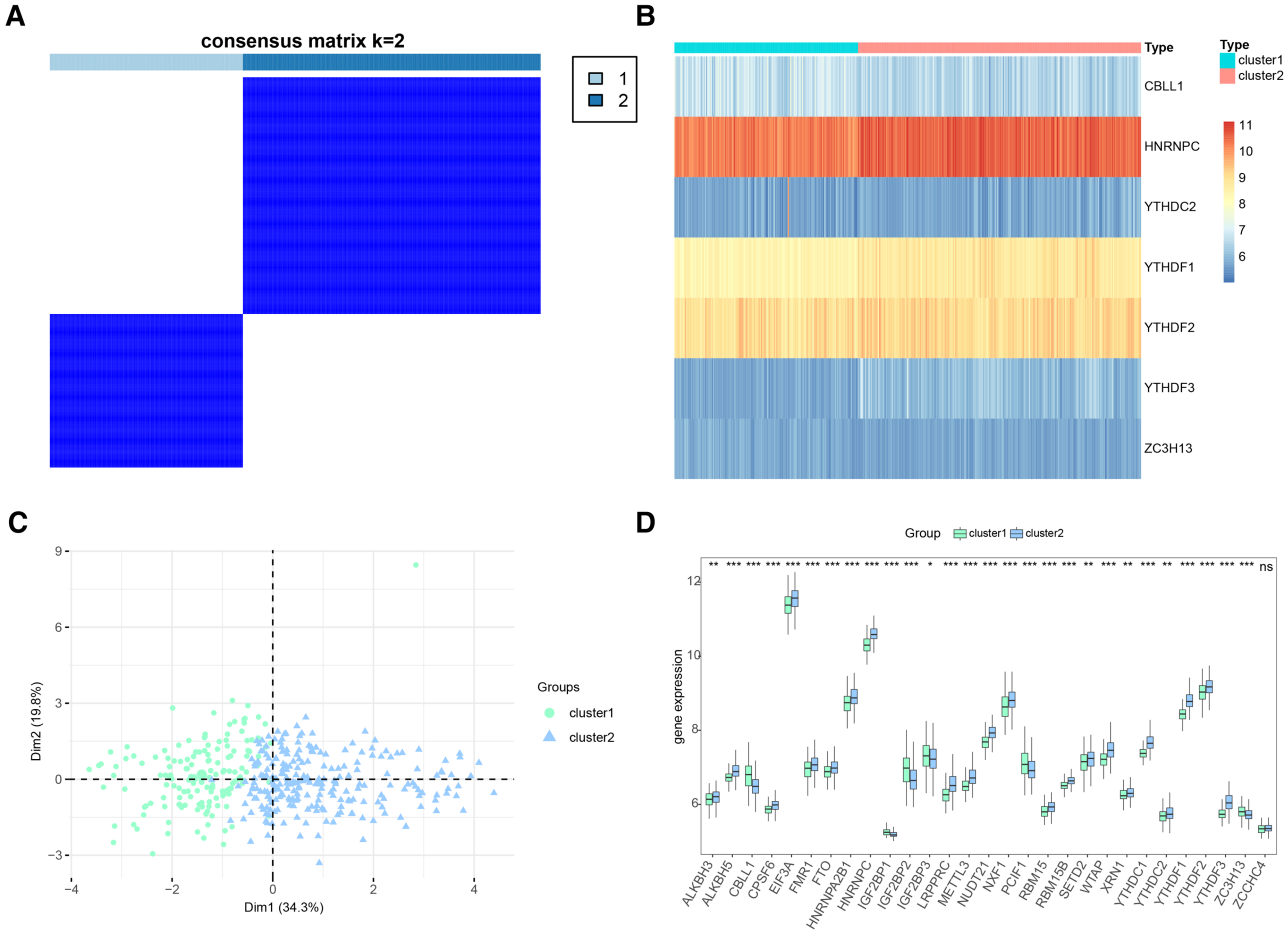
Consensus clustering of significant RNA N6-methyladenosine (m6A) regulators in CHD patients. (**A**) Consensus matrix of the 7 significant m6A regulators for k = 2. (**B**) Expression heat map of the 7 significant m6A regulators in cluster1 and cluster2. (**C**) Principal component analysis for the expression profiles of the 7 significant m6A regulators that shows a remarkable difference in transcriptomes between the two patterns. (**D**) Differential expression histogram of the 7 significant m6A regulators in cluster1 and cluster2.

Based on the 7 significant genetic results collected by our regression model model, we implemented a consensus clustering approach to identify distinct m6A clusters based on the 7 key m6A regulators. This process was conducted using the “ConsensusClusterPlus” package in R software, and it led to the identification of two discrete m6A clusters (Figure 8A). Through our consensus clustering analysis, we found that cluster1 comprised 157 individuals, while cluster2 was larger and included 242 individuals. To illustrate the distinct m6A regulator expression patterns across the two clusters, we created both a heat map and a histogram. This graphical representation provided a clear visual distinction between the two clusters in terms of m6A regulator expression levels (Figure 8B). Further analysis using principal component analysis (PCA) revealed that the 10 crucial m6A regulators were able to effectively separate these two m6A clusters, demonstrating the significant differences in their m6A regulation profiles (Figure 8C). We observed a noticeable difference in the expression of various m6A regulators between the two clusters. Specifically, ALKBH3, RBM15B, RBMX, SETD2, ALKBH5, CPSF6, EIF3A, FMR1, FTO, HNRNPA2B1, HNRNPC, YTHDC1, YTHDC2, YTHDF1, YTHDF2, LRPPRC, METTL14, PRRC2A, RBM15, SRSF10, SRSF3, TRMT112, VIRMA, WTAP, XRN1, METTL16, METTL3, NUDT21, NXF1, YTHDF3, and ZC3H13 all demonstrated lower expression levels in cluster1 compared to cluster2. Conversely, CBLL1, IGF2BP2, IGF2BP3, IGF2BP1, and PCIF1 showed higher expression in cluster1 than in cluster2 (Figure 8D).

These findings highlight the existence of distinct m6A methylation patterns among individuals with CHD, suggesting that differences in the expression of key m6A regulators could be linked to variations in disease progression or response to treatment. Further research is necessary to validate these findings and explore their potential implications for the management and treatment of CHD.

### Differential analysis of Two distinct m6A gene clusters

**Figure 9:**
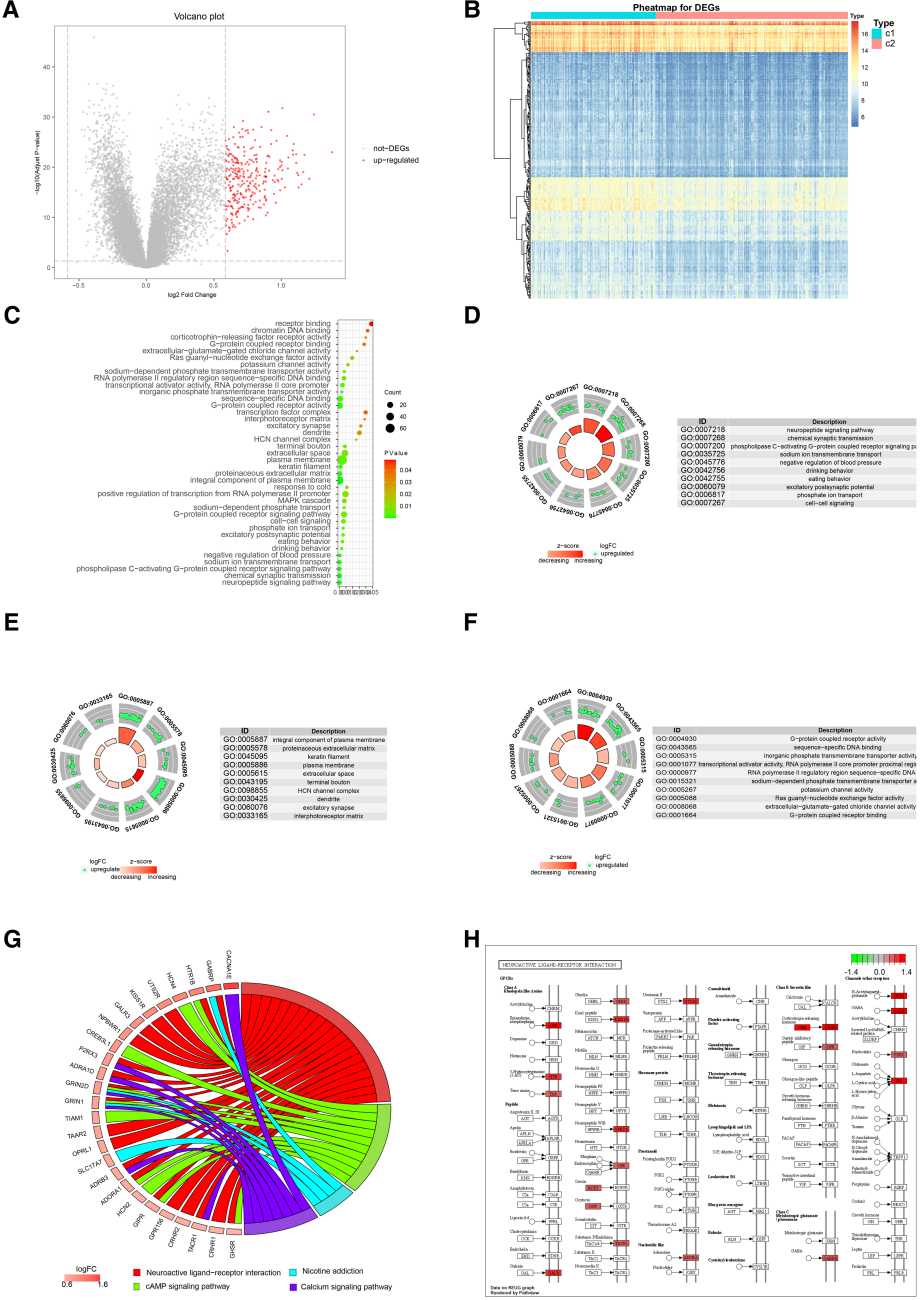
Go and KEGG analysis of two distinct m6A regulator clusters. (**A**) 277 up-regulated genes were selected (red nodes). (**B**) Expression heat map of 277 DEGs in cluster1 and cluster2. (**C**) GO enrichment analysis of 277 up-regulated genes. (**D**) Presentation of top 10 biological process. (**E**) Presentation of top 10 cellular component. (**F**) Presentation of top 10 molecular function. (**G**) Presentation of KEGG pathway analysis. (**H**) Details of significant enrichment in hsa04080: Neuroactive ligand-receptor interaction.

**Figure 10:**
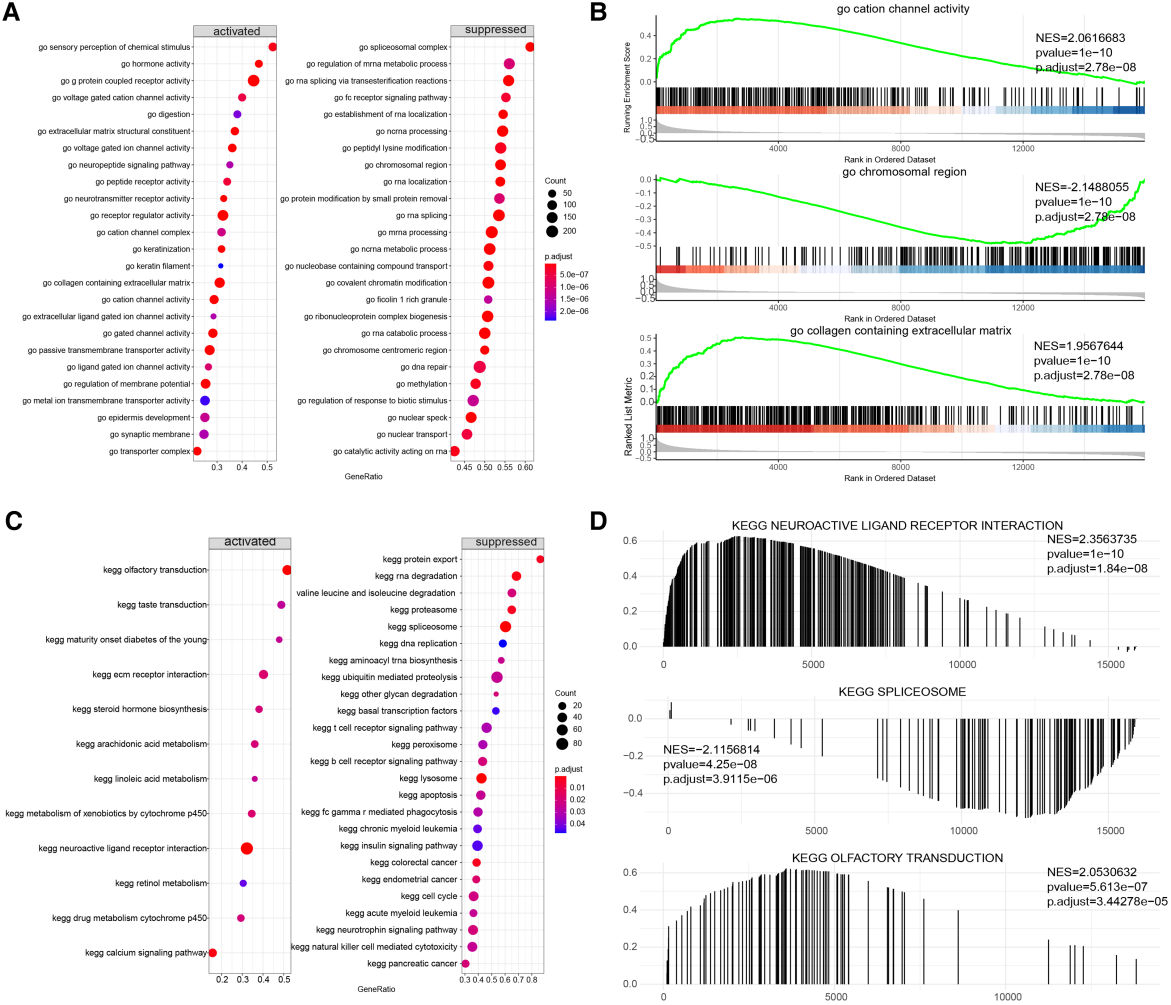
Gene set enrichment analysis (GSEA) of two distinct m6A regulator clusters. (**A,B**) GSEA-GO analysis of two distinct m6A regulator clusters. (**C,D**) GSEA-KEGG analysis of two distinct m6A regulator clusters.

To further explore the potential functional differences between the two clusters defined by the m6A regulators, we selected 277 significant m6A-related DEGs (Figure 9A). A heatmap demonstrated that these DEGs effectively explained the differentiation of expression between the two clusters (Figure 9B). For functional annotation of the biological differences between the clusters, GO analysis was performed on these DEGs (Figure 9C) (Supplementary file 1). This analysis indicated that the candidate genes were associated with various biological processes such as neuropeptide signaling pathway, chemical synaptic transmission, phospholipase C-activating G-protein coupled receptor signaling pathway, sodium ion transmembrane transport, and negative regulation of blood pressure (Figure 9D). They were also involved in cellular components such as integral components of plasma membrane, proteinaceous extracellular matrix, keratin filament, plasma membrane, and extracellular space (Figure 9E). In terms of molecular functions, these genes showed significant enrichment in functions like G-protein coupled receptor activity, sequence-specific DNA binding, inorganic phosphate transmembrane transporter activity, transcriptional activator activity, and RNA polymerase II core promoter proximal region sequence-specific binding (Figure 9F). KEGG pathway analysis revealed that the DEGs were significantly enriched in pathways like Neuroactive ligand-receptor interaction, cAMP signaling pathway, Nicotine addiction, and Calcium signaling pathway (Figure 9G). We further visualized the details of DEGs involved in the Neuroactive ligand-receptor interaction pathway (Figure 9H) (Supplementary file 2). Using GSEA, we analyzed all the genes across the two clusters. Our results suggested that in cluster1, biological processes like keratinization, G-protein coupled receptor activity, hormone activity, neurotransmitter receptor activity, extracellular ligand-gated ion channel activity, and others were activated, whereas processes like spliceosomal complex formation, peptidyl lysine methylation, RNA localization establishment, RNA splicing, and others were suppressed (Figures 10A,B). In contrast, in cluster2, biological processes like neuroactive ligand receptor interaction, olfactory transduction, maturity onset diabetes of the young, linoleic acid metabolism, steroid hormone biosynthesis, and others were activated, while processes like spliceosome formation, protein export, proteasome activity, lysosome activity, RNA degradation, and others were inhibited (Figures 10C,D) (Supplementary files 3, 4).

These results provide essential insights into the intricate roles played by the two clusters of m6A methylation regulators, which appear to be intricately associated with CHD's biological processes and pathways. Each cluster exhibited a unique functional profile, indicative of differing pathophysiological mechanisms in CHD, and this disparity might underlie variations in disease progression and patient outcomes.

### Immune characteristic of Two distinct m6A gene clusters

In an attempt to further characterize the two clusters and elucidate the potential mechanisms driving their unique functional profiles, we turned our attention to the immune microenvironment. We employed two powerful computational tools -ssGSEA and CIBERSORT—to estimate the level of immune cell infiltration in the overall sample and in both clusters.

**Figure 11:**
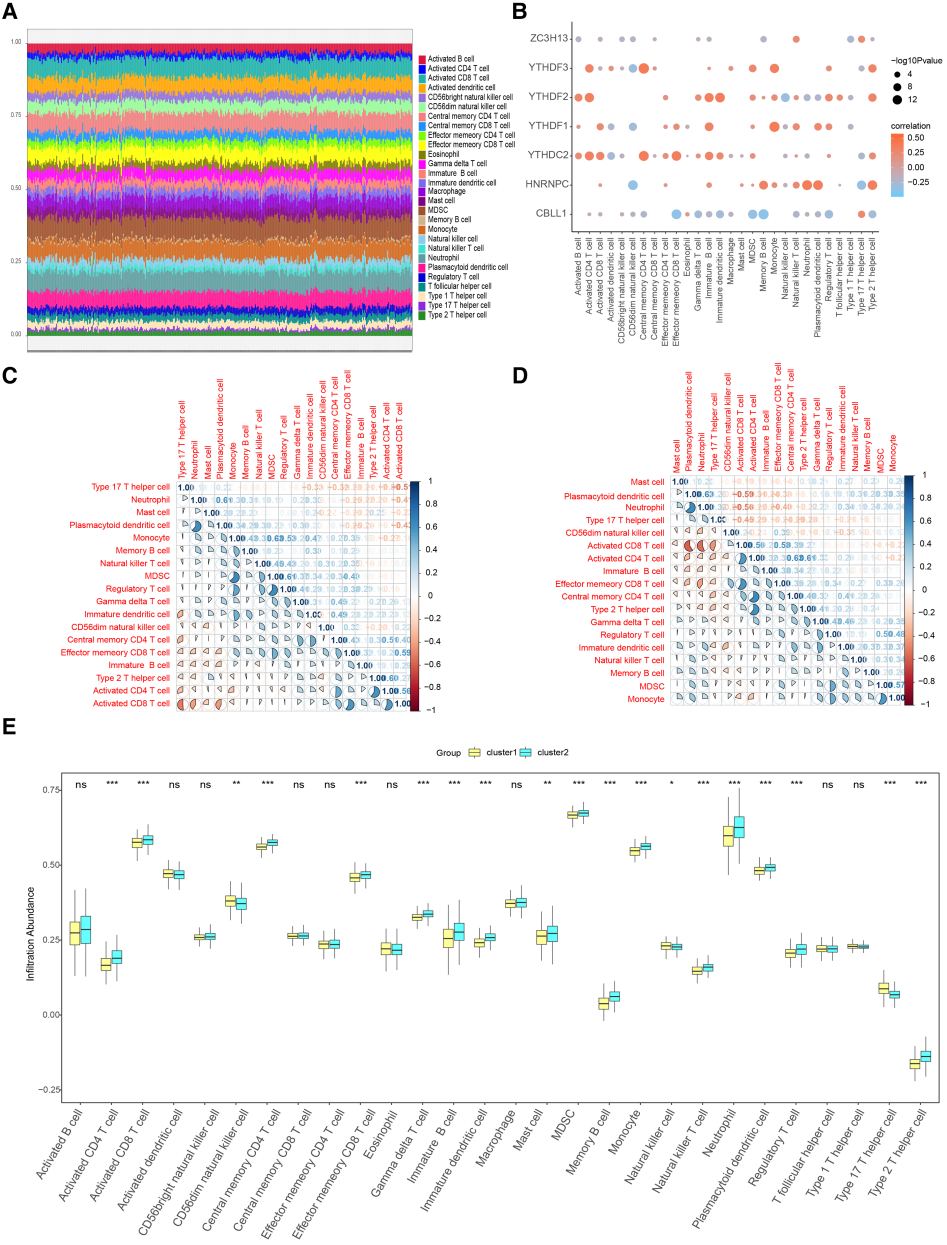
Immune characteristic analysis by single sample GSEA of two distinct m6A gene clusters. (**A**) Immune cell contents of two clusters, horizontal axis represents identities of patients. (**B**) Correlation between 7 m6A regulators and immune cells. (**C**) Correlation between 7 m6A regulators and immune cells in Cluster1. (**D**) Correlation between 7 m6A regulators and immune cells in Cluster2. (**E**) Histogram of immune cell contents in two clusters.

The results of the ssGSEA were shown Figure 11A. We further explored the correlation between immune cells and seven important genes in the overall sample and found that all of them, except ZC3H13 and CBLL1, showed significant positive correlation with most of the immune cells (Figure 11B). Additional, to further explore the correlation of seven important genes with the outcome of immune infiltration by different clusters. Our correlation analysis revealed several interesting interactions among immune cell types. In cluster1, we noted a negative correlation between activated CD8 T cells and Type 17 T helper cells, suggesting that the activation of these two cell types might be mutually exclusive within this cluster. Additionally, neutrophils and plasmacytoid dendritic cells exhibited positive correlations with Myeloid-derived suppressor cells (MDSCs), hinting at a potential cooperative relationship in driving CHD pathology (Figure 11C). Contrastingly, in cluster2, activated CD8 T cells and plasmacytoid dendritic cells negatively correlated with Type 17 T helper cells. Neutrophils, plasmacytoid dendritic cells, and activated CD4 T cells all showed positive correlations with activated CD8 T cells, suggesting a different immunological interplay within this cluster (Figure 11D). Importantly, Figure 11E showed a significant reduction of immune cell content in cluster1 when compared to cluster2. This finding suggests a potential immunosuppressive environment in cluster1, which might be conducive to CHD progression.

**Figure 12:**
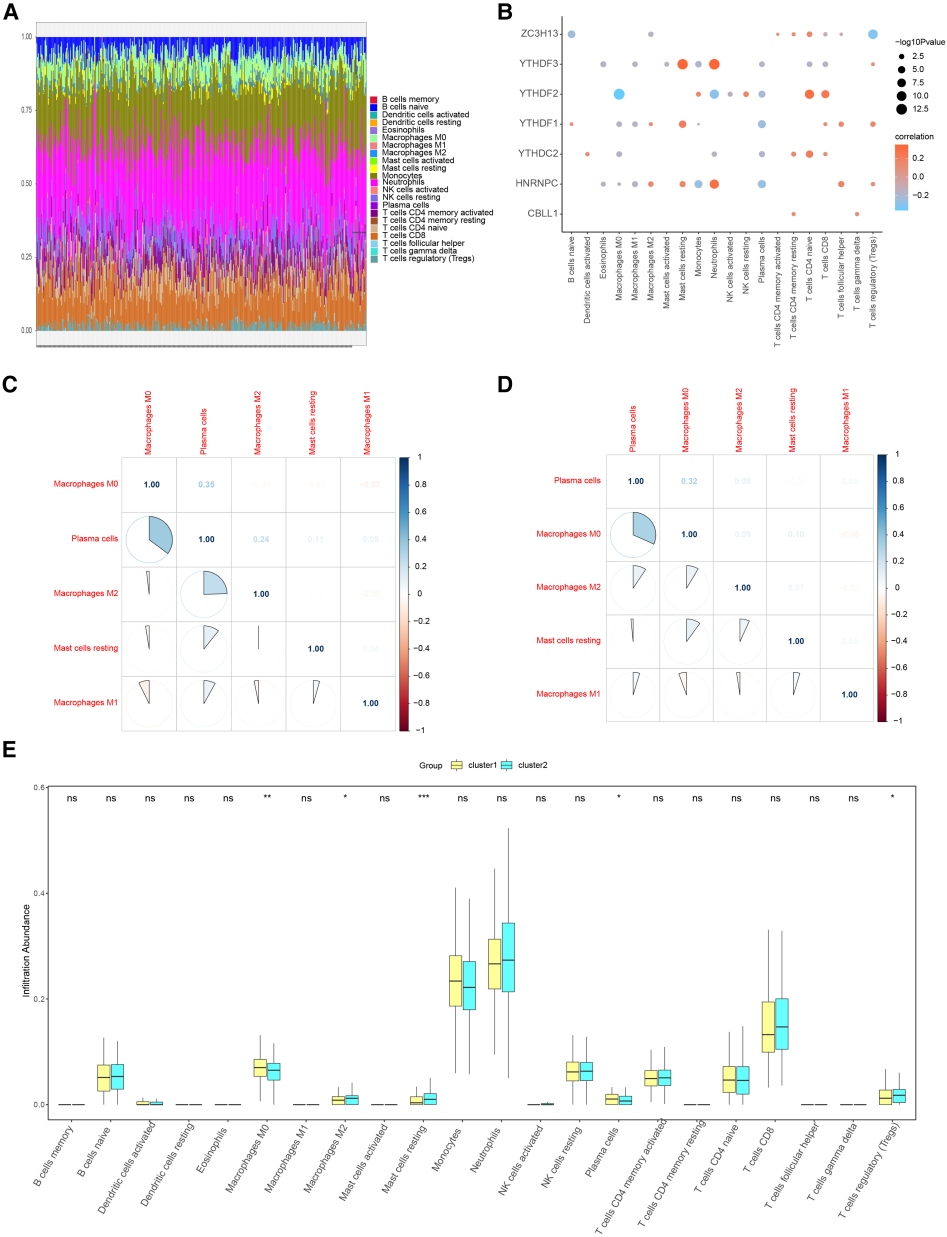
Immune characteristic analysis by CIBERSORT of two distinct m6A gene clusters. (**A**) Immune cell contents of two clusters, horizontal axis represents identities of patients. (**B**) Correlation between 7 m6A regulators and immune cells. (**C**) Correlation between 7 m6A regulators and immune cells in Cluster1. (**D**) Correlation between 7 m6A regulators and immune cells in Cluster2. (**E**) Histogram of immune cell contents in two clusters.

When we examined the correlation between the 7 candidate genes and immune cell content, we found a close association. This implies that these genes might exert their influence on CHD development partly through modulating immune cell activity and infiltration. We further corroborated these findings using the CIBERSORT algorithm. Figure 12A shows the results of CIBERSORT in the overall sample. Importantly, our analysis affirmed a close relationship between the seven candidate regulators and immune cell content in the overall sample and the results obtained by ssGSEA were similar (Figure 12B). To further explore the correlation of seven important genes with the outcome of immune infiltration by different clusters, we found Plasma cells were found to negatively correlate with M0 macrophages (Figures 12C,D), suggesting distinct roles for these cell types in shaping the immune landscape in the clusters.

Similar to ssGSEA, a marked reduction in immune cell content was detected in cluster1 relative to cluster2 (Figure 12E).

These results offer more compelling evidence that m6A methylation regulators may influence CHD progression through their impact on immune cell dynamics and the immune microenvironment. In summary, our results underscore the critical role of immune infiltration in CHD's pathogenesis and its potential interaction with m6A methylation regulators. This provides a new perspective for understanding the disease mechanism and identifying novel therapeutic targets.

### Diagnostic efficiency and drug prediction of 7 candidate regulators

**Figure 13:**
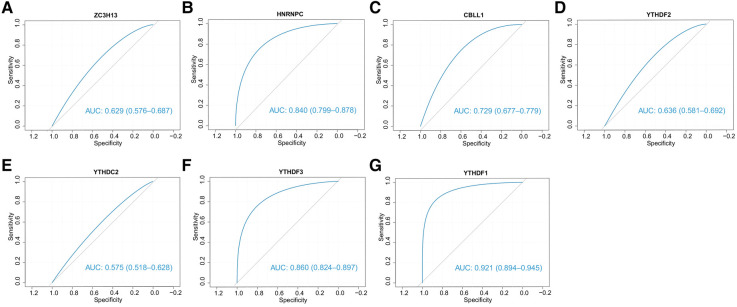
The ability of 7 candidate regulators to distinguish two clusters. The ROC curves showed that two clusters can be stably distinguished by 7 regulators.

**Figure 14:**
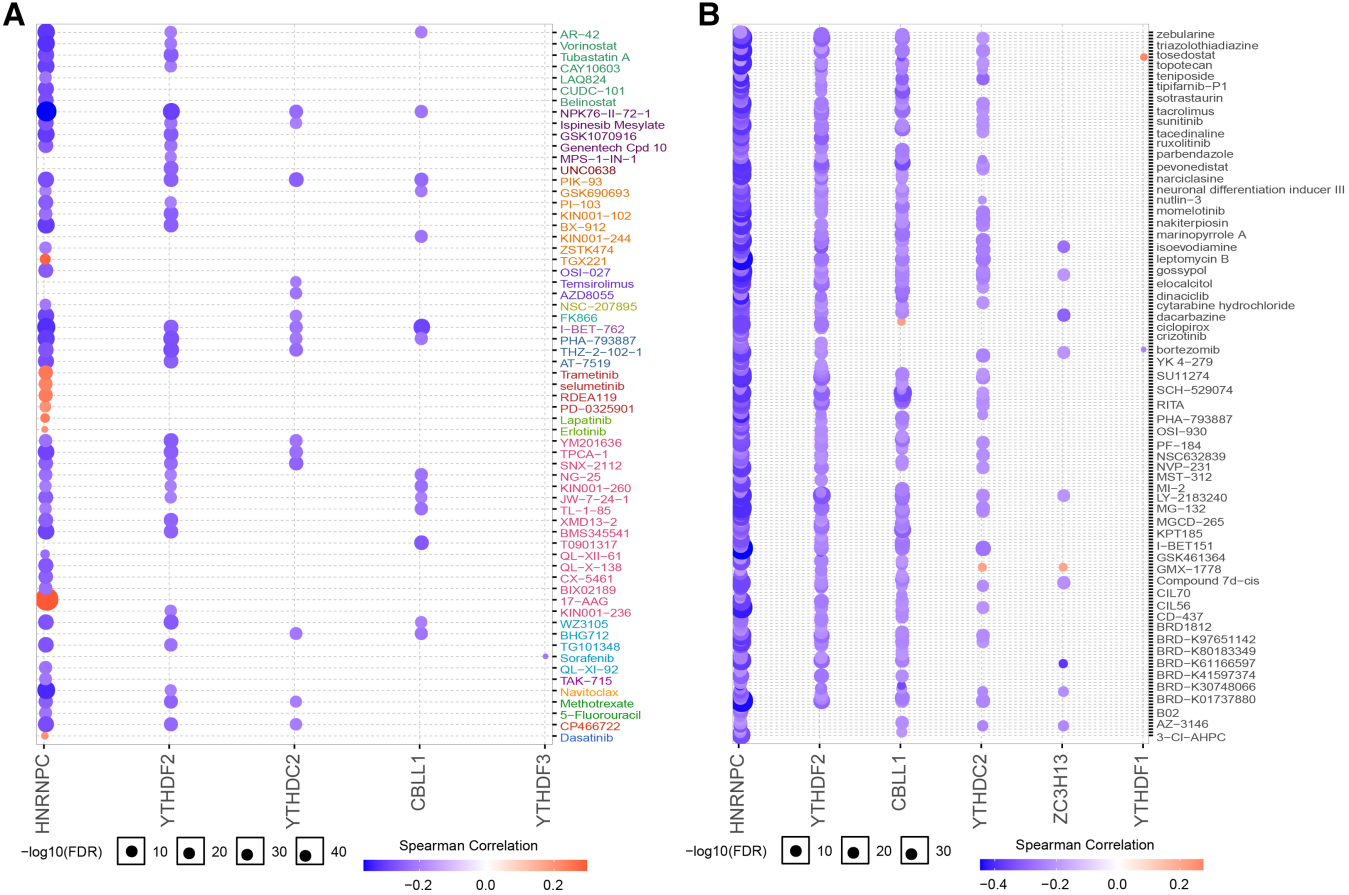
Drug sensitivity analysis of 7 regulators. (**A**) Regulator gene expression levels and drug sensitivity were measured by Genomics of GDSC. (**B**) Regulator gene expression levels and drug sensitivity were measured by CTRP.

We subsequently assessed the discriminative ability of the 7 candidate m6A regulators to distinguish between the two clusters. The ROC curves demonstrated that each of the candidate regulators indeed significantly differentiated cluster1 from cluster2 (Figures 13A–G). This implies that these regulators not only contribute to the unique characteristics of each cluster but also may serve as potential biomarkers for the classification of CHD patients into different risk or pathological categories. Furthermore, we explored the potential therapeutic implications of these 7 candidate regulators by predicting their drug sensitivity. Our findings revealed that these regulators are implicated in various diseases as drug targets. This suggests their potential for wide therapeutic applicability. More importantly, they exhibited high sensitivity to several drugs (Figures 14A,B). The drug significantly positively associated with HNRNPC was 17-AAG, the drug significantly positively associated with CBLL1 was dacarbazine, the drug significantly positively associated with YTHDC2, ZC3H13 was GMX-1778, and the drug significantly positively associated with YTHDF1 was tosedostat. However, these drugs were not found to be associated with CHD, and further exploration is needed.

Taken together, our results provide evidence for the involvement of m6A regulators in the pathogenesis of CHD and suggest potential therapeutic strategies targeting these regulators. By doing so, it is hoped that new avenues may be paved for the development of precision medicine for CHD, tailored to the individual patient's genetic makeup and disease subtype.

### Expression validation of 7 diagnostic m6A regulators

**Figure 15:**
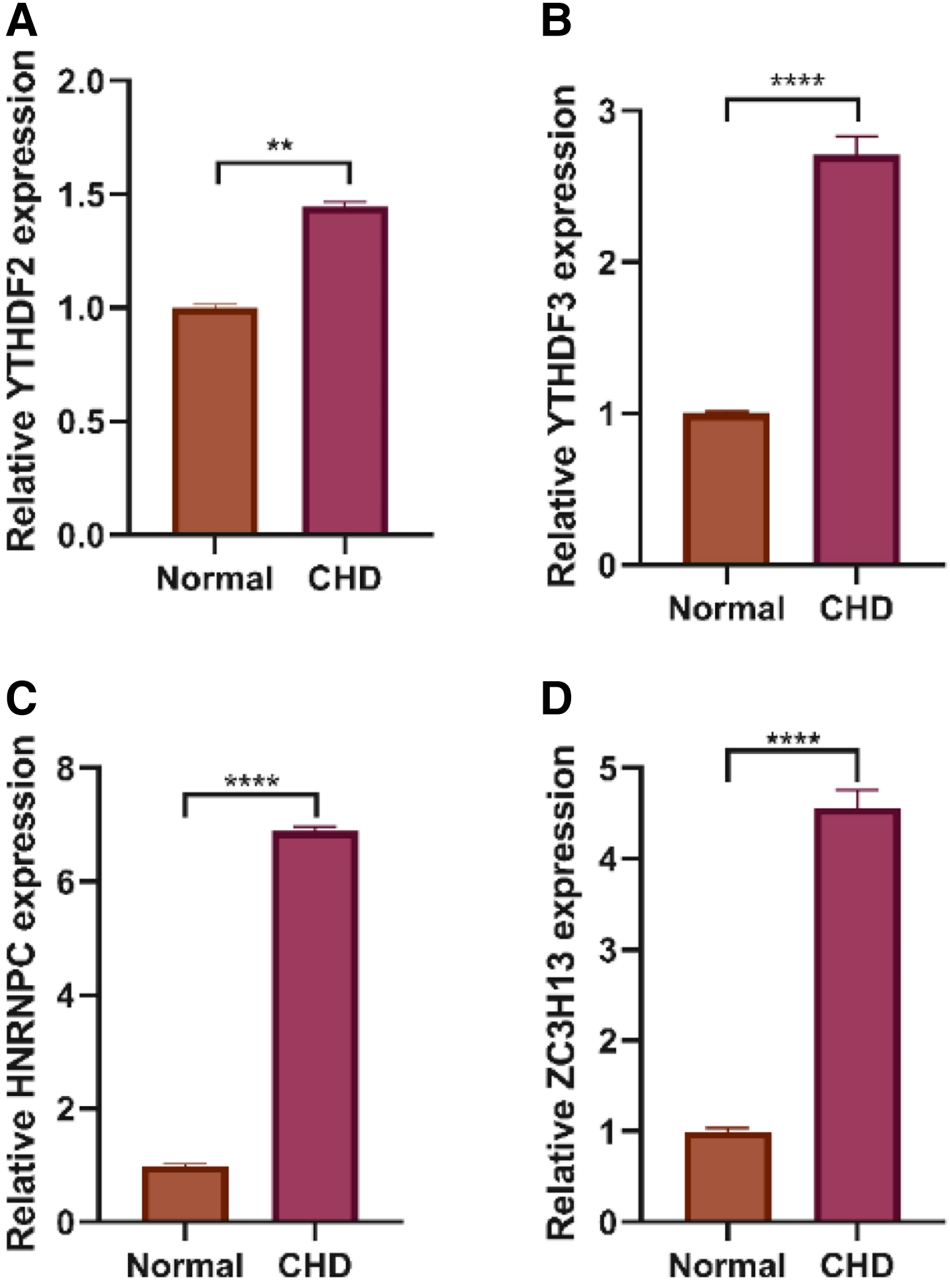
Expression levels of 4 m6A genes between CHD and normal tissues. (**A**) YTHDF2 was significantly highly expressed in CHD tissues. (**B**) YTHDF3 was significantly highly expressed in CHD tissues. (**C**) HNRNPC was significantly highly expressed in CHD tissues. (**D**) ZC3H13 was significantly highly expressed in CHD tissues. Results of quantitative real-time PCR for the 4 m6A genes. The expression of hub genes was standardized relative to the expression of GAPDH. The significance of differences was determined using the student's *t*-test; *P < 0.05, **P < 0.01, ***P < 0.001, ****P < 0.0001.

We collected 4 normal and 4 CHD blood samples from Affiliated People's Hospital of Jiangsu University and elucidated the expression changes of selected diagnostic mRNAs in CHD by qRT-PCR. Four m6A regulators were significantly differentially expressed between CHD and normal samples. The expression levels of YTHDF2, YTHDF3, ZC3H13 and HNRNPC were remarkably up-regulated in CHD samples compared with normal samples (Figures 15A–D).

## Discussion

Despite improvements in prevention, diagnosis, and early management, CHD remains a leading cause of death and disability worldwide (1). Evidence suggests that m6A regulators play a critical role in a myriad of cellular and organismal processes (21). However, the role of m6A regulators in CHD is still unclear. Our study aimed to understand better how m6A regulators contribute to CHD.

Initially, we compared the expression levels in individuals without CHD to those with CHD, identifying 37 potential m6A regulators. Among these, seven—CBLL1, HNRNPC, YTHDC2, YTHDF1, YTHDF2, YTHDF3, and ZC3H13—were selected as potential predictors of CHD prevalence using logistic regression analysis. We analyzed the correlation between writers and erasers in CHD patients. Due to the scarcity of m6A regulators in the public database, we were unable to independently verify our model. We used these seven potential m6A regulators to create a nomogram model, and the DCA curve showed that nomogram-based decisions correlated with better outcomes for CHD patients.

CBLL1, also known as E3 ubiquitin-protein ligase Hakai, possesses a RING-finger domain involved in the ubiquitination, endocytosis, and degradation of herin and plays a significant role in TNF or cytokine reactions in periodontitis (21), is a significant regulator in TNF or cytokine reactions in periodontitis (22). HNRNPC, an m6A reader protein, binds m6A-modified RNA via an “m6A-switch” mechanism, in which the instability of an RNA hairpin facilitated by m6A unveils a single-stranded HNRNPC binding motif (23). HNRNPC may be associated with the malignant development of glioblastoma multiforme and be predictive of a favorable prognosis (24). Moreover, HNRNPC may influence to tumorigenesis by altering expression of genes that modulate cell proliferation and metastasis (25). YTHDC2 can promote the translation of target mRNAs (21), and it may serve as an activator in colon cancer metastasis and could be a diagnostic marker for individuals with colon cancer (26). YTHDF1, YTHDF2, and YTHDF3 proteins, found predominantly in the cytoplasm (27) bind to m6A via their C-terminal YTH domains (28). YTHDF1 and YTHDF3 can enhance the translation of a small set of m6A mRNAs. YTHDF1 augments translation efficiency by interacting with members of the eIF3 complex (29) and plays a critical oncogenic role (27, 30). YTHDF2, on the other hand, induces mRNA degradation by binding to the m6A modification site (31) and is significantly increased in pancreatic cancer tissues, with levels much higher in patients with advanced disease (32). ZC3h13 is a zinc-finger protein (33). A study has shown that ZC3h13 is essential for m6A methylation, and depletion of ZC3h13 predominantly affects m6A methylation at the 3′ UTR of mRNA (34). ZC3h13 may play a crucial role in breast cancer (35). Several studies have shown that the seven potential m6A regulators are involved in the incidence and progression of cancers, affecting proliferation, invasion, radiation resistance, and prognosis (35–37). However, no published studies have explored the connection between these seven potential m6A variants and CHD. We believe our study could provide insight for future experimental research.

Our risk prediction model has demonstrated good performance in predicting the occurrence of CHD. However, further experimental validation is needed to support our findings before the model can be widely used for clinical applications. The CHD nomogram allows for a more intuitive individual risk assessment, providing a numerical probability that can help clinicians decide the timing of potential preventative interventions (38, 39).

While genomes and epigenomes of CHD have been available for over a decade, many of the genes have yet to be characterized in terms of their functions. By identifying clusters of genes with highly similar expression profiles, we can infer the function of uncharacterized genes from their neighboring genes (40). Previous research has shown that the arrangement of genes in genome clusters can significantly affect biological processes (41). The two identified m6A gene clusters suggest that certain biological processes might be influenced in CHD. To explore this further, we employed GO, KEGG, and GSEA analysis. Our findings suggest that G-protein coupled receptor activity and neuroactive ligand-receptor interaction may play vital roles in CHD.

It is now believed that the pathophysiology of CHD is strongly connected to different subsets of the T cell population (42, 43). Distinct influences on atherosclerosis have been observed from different subgroups of CD4+ T cells. While Th1 cells are thought to contribute to the development of atherosclerosis, the role of Th2 cells remains controversial (42). Tregs are considered beneficial in CHD due to their production of transforming growth factor and IL10; adoptive transfer of Tregs has been demonstrated to minimize atherosclerosis, while depletion of Tregs increases atherosclerosis (42, 44). Atherosclerotic changes are partly caused by the activation of cells such as neutrophils, monocytes, macrophages, and dendritic cells (45). Therefore, targeting the immune cells involved in atherogenesis represents a promising new approach for atherosclerosis treatment and prevention (46). In our study, two clusters exhibited different immunological characteristics, suggesting that precise immunotherapy and targeted therapy could be tailored based on these characteristics.

Finally, we assessed the drug sensitivity of the seven candidate regulators. Accurate predictions can save time and resources in drug discovery and development (47), and a thorough understanding of drug mechanisms of action can shed light on how drugs work (48).

## Conclusion

In conclusion, we identified seven candidate m6A regulators and developed a nomogram to predict the occurrence of CHD. Using the seven important m6A regulators, we discovered two m6A clusters and suggested innovative treatments for coronary disease based on these clusters.

## Data Availability

The original contributions presented in the study are included in the article/Supplementary Material, further inquiries can be directed to the corresponding authors.
